# Fluorescence-illuminated Diffraction Tomography using Explicit Neural Fields

**DOI:** 10.21203/rs.3.rs-6442385/v1

**Published:** 2025-05-23

**Authors:** Yi Xue, Renzhi He, Yucheng Li, Junjie Chen

## Abstract

Multimodal imaging of fluorescence and phase provides distinct and complementary insights into biological samples. Current multimodal techniques for simultaneous phase and fluorescent imaging primarily operate in transmission mode and are limited to thin samples, restricting their applications to bulky tissues and in vivo animals. While multiphoton microscopy has enabled deeptissue fluorescence imaging, integrating it with phase imaging remains challenging due to the limited availability of methods capable of reconstructing the 3D refractive index (RI) of bulky, label-free tissues in reflection mode at subcellular resolution. To bridge the technical gap, we develop fluorescence-illuminated diffraction tomography (FDT) that reconstructs the 3D RI of label-free objects from diffracted fluorescence images acquired in reflection mode under two-photon excitation. The RI reconstruction leverages the transport of intensity equation (TIE) and is solved by a self-supervised neural network based on explicit neural fields. Compared to the state-of-the-art implicit neural fields, the explicit neural fields significantly improve computational speed, reconstruction accuracy, and interpretability. Using FDT, we successfully reconstruct the 3D RI of a 300 µmthick label-free bovine myotube sample over a 530 × 530 µm2 field-of-view at subcellular resolution within 20 min. FDT is the first technique to extract 3D RI from diffracted fluorescence images in reflection mode for thick tissues, overcoming key limitations of existing multimodal systems. This work lays the foundation for broadly accessible, reflection-mode multimodal fluorescence-phase imaging in complex biological systems in the future.

## Introduction

Multimodal microscopy for fluorescence and phase imaging provides distinct and complementary signals of biological samples [1–13]. Fluorescence microscopy provides molecular specified information of the sample by imaging intrinsic or exogenous fluorescence, while quantitative phase microscopy measures the refractive index (RI) of the sample, which can be used as a biomarker for cell biomechanical properties and mass transportation. Most multimodal microscopy techniques for fluorescence and phase imaging are in transmission mode and designed for imaging thin transparent samples [1–11]. In these techniques, quantitative phase imaging of 3D refractive index (RI) is acquired by optical diffraction tomography (ODT) [14, 15] or intensity diffraction tomography (IDT) [16, 17], while fluorescence images are acquired by widefield illumination [18–23]. These techniques are in transmission mode because, in phase imaging, 90% of the illumination light is forward-scattered. As a result, detecting forward-scattered light in transmission mode achieves a much higher signal-to-noise ratio (SNR) compared to phase imaging in reflection mode, which relies on back-scattered light. On the other hand, fluorescence emission is isotropic with no preference for forward or backward scattering.

However, the transmission geometry of these multimodal fluorescence-phase microscopy limits their applications on imaging bulky tissue or in vivo animals, where transmitted photons are very few or the transmission side of the sample is not accessible. As the thickness of tissue increases, the number of photons propagating through the bulky tissue decreases exponentially, and the number of fluorescence or back-scattered photons after propagating back through the bulky sample is even fewer. The other challenge for multimodal fluorescence-phase imaging of bulky tissue is optical sectioning to distinguish signals from a specific deep layer while suppressing background from other depths. For reflection-mode phase imaging, optical sectioning ability has been achieved by using low temporal coherence light sources [24], confocal detection [25], and interference [26, 27]. For fluorescence microscopy, optical sectioning ability has been achieved by confocal detection [28], multiphoton excitation [29], and structured illumination [30]. However, these reflection-mode phase imaging techniques are incompatible with fluorescence imaging simultaneously using the same setup, since the difference of illumination sources could cause severe crosstalk if both are turned on concurrently.

Enabling simultaneous fluorescence and phase imaging in reflection mode requires a new phase imaging technique for 3D RI measurement in bulky tissue and compatible with fluorescence imaging for deep tissue. Our recent work, bi-functional refractive index and fluorescence (BRIEF) microscopy [12], enables simultaneous fluorescence and phase imaging by decoding both fluorescence and phase signals from diffracted fluorescence images. Instead of sequentially capturing fluorescence and phase images of labeled and label-free structures, BRIEF leverages excited sparsely-distributed fluorescent beads as internal illumination sources within the sample. As fluorescence propagates through label-free tissue, it encodes the tissue's heterogeneous RI in the resulting diffracted fluorescence images. The 3D RI and fluorescence intensity are then reconstructed by solving an inverse problem using a gradient decent optimization algorithm. BRIEF establishes a proof-of-concept for simultaneous, multimodal fluorescence and phase imaging of thin, transparent samples with sparsely-distributed fluorescent beads.

However, applying BRIEF to image bulky tissue with dense fluorescence expression introduces substantial challenges in both hardware and software. For densely labeled fluorescence samples, new optical hardware is required to generate a single diffraction-limited focus at a time as the point-source illuminator inside the sample, preventing crosstalk from multiple illuminators and preserving the contrast of diffracted images. Resolving the 3D RI of bulky tissue requires improved axial resolution and $z$-sectioning capability, which necessitates a large number of measurements from wide oblique angles. To achieve accurate 3D RI reconstruction of bulky tissue, a new algorithm is needed to efficiently process the large dataset, jointly estimate fluorescence illumination positions, and account for the partially coherent/incoherent nature of fluorescence. Although partially coherent/incoherent fluorescence can theoretically be modeled using the Wigner distribution function [31, 32], applying this approach to fluorescence from individual fluorophores is impractical due to case-by-case variations in coherence, which depend on the fluorophore type, the spatial focus of two-photon excitation, and tissue scattering. Therefore, the new algorithm should incorporate a more practical strategy to handle fluorescence images effectively. Furthermore, bulky tissue causes multiple diffraction of fluorescence, reducing image contrast and increasing the computational complexity of 3D RI reconstruction. The new algorithm must address both the multiple diffraction and the low signal-to-noise ratio of diffracted fluorescence images.

To tackle these challenges, we developed fluorescence-illuminated diffraction tomography (FDT) that reconstructs the 3D RI of bulky tissue using explicit neural fields from two-photon fluorescence images acquired in reflection mode. Here, "explicit neural fields" are a class of deep learning models [33] that represent 3D object using grid-based structures combined with neural networks, unlike implicit neural fields [34–36] that encode the object as a continuous function. Implicit neural fields combined with physics-based forward models have been broadly used in microscopic imaging, such as such as phase recovery [37] ptychographic microscopy [36], volumetric fluorescence imaging [38], adaptive optics [39], wavefront shaping [40], and dynamic structured illumination [41]. However, the implicit methods rely on the encoding/embedding methods, such as positional encoding [34], hash encoding [41] and radial encoding [37] to learn the high-frequency information [42], particularly when reconstructing dense and complex scenes. Moreover, the computational speed of implicit neural fields is relatively slow, limiting its scalability for processing large datasets. Our data demonstrate that explicit neural fields not only accelerate computation but also deliver significantly enhanced reconstruction quality. Therefore, we use explicit neural fields for high-resolution 3D reconstruction of RI. More importantly, we upgrade the explicit neural fields with four key innovations, including a coarse-to-fine strategy for alias-free 3D reconstruction, a self-calibration of illumination source for precise reconstruction, a multi-slice model to render light propagation, and a partially coherent mask to reduce the modal mismatch between the experimental data and the theoretical model. To lift the restriction on sparse expression of fluorescence without causing crosstalk, we implement two-photon excitation instead of one-photon excitation in

BRIEF to generate diffraction-limited focus and optical sectioning in densely labeled, highly scattering fluorescent samples.

We demonstrate, for the first time, that FDT can recover the 3D RI of bulky 3D-cultured bovine myotubes up to ∼300 $\mu m$ thick with subcelluar resolution using reflection-mode fluorescence images. We also quantitatively compare FDT using explicit neural fields to FDT using implicit neural fields by reconstructing the 3D RI of silica beads and living Madin-Darby Connie Kidney (MDCK) cells (∼44 $\mu m$ thick) from experimentally acquired images. By integrating fluorescence excitation with IDT, FDT provides a unique approach for reconstructing the 3D RI of bulky tissue from fluorescence images. FDT is a critical step toward multimodal fluorescence and phase microscopy for bulky tissue and ultimately for in vivo imaging in the future.

## Results

### Optical setup of FDT

FDT acquires diffracted fluorescence images excited by two-photon excitation, enabling the generation of diffraction-limited fluorescent focus (the new "fluorescent illumination source") within the sample regardless of the density of fluorescence label distribution. The excitation path of the optical setup is the same as a standard point-scanning two-photon microscope (Fig. 1e). A femtosecond pulsed laser beam at 1035 nm is collimated and scanned in the $x$-$y$ plane by a pair of galvo mirrors, and in the $z$ direction by an electrically tunable lens. The galvo mirrors and the electrically tunable lens can be functionally replaced by a spatial light modulator for beam steering, as both approaches achieve the same control of light in 3D (Fig. 1e, inset). The innovation of FDT hardware lies in the detection path. Instead of using a single-pixel detector (e.g., a photomultiplier tube) to collect two-photon images at the focal plane, FDT uses a camera to capture *diffracted* two-photon images on a *defocused* plane at each scanning location (Fig. 1c). The size of the area illuminated by each fluorescent illumination source in the defocused fluorescence images (Fig. 1b) is proportional to the depth of the source. These diffracted images encode the RI of label-free structures located above the fluorescent illumination source as the emitted fluorescence propagates through them. Capturing the fluorescence images on a defocused plane enables 3D RI reconstruction using the transport of intensity equation (TIE) [43–45].

### Model architecture of FDT

FDT reconstructs 3D RI from diffracted fluorescence images in four steps (Fig. 1a-d). First, we developed a coarse-to-fine process to avoid aliasing. In the process, a 3D grid explicitly represents the unknown 3D RI and controls the granularity of the RI, which is gradually increased as the number of iterations increases (details in Methods 1). The unknown RI is set as the parameters to be optimized within the PyTorch framework. We compare the reconstructed 3D RI with and without the coarse-to-fine process from simulated data (Fig. 1a). The result (letter "i") shows the coarse-to-fine process successfully solve both low and high spatial frequency components, compared to the aliasing result without the coarse-to-fine process. It improves the MSE between

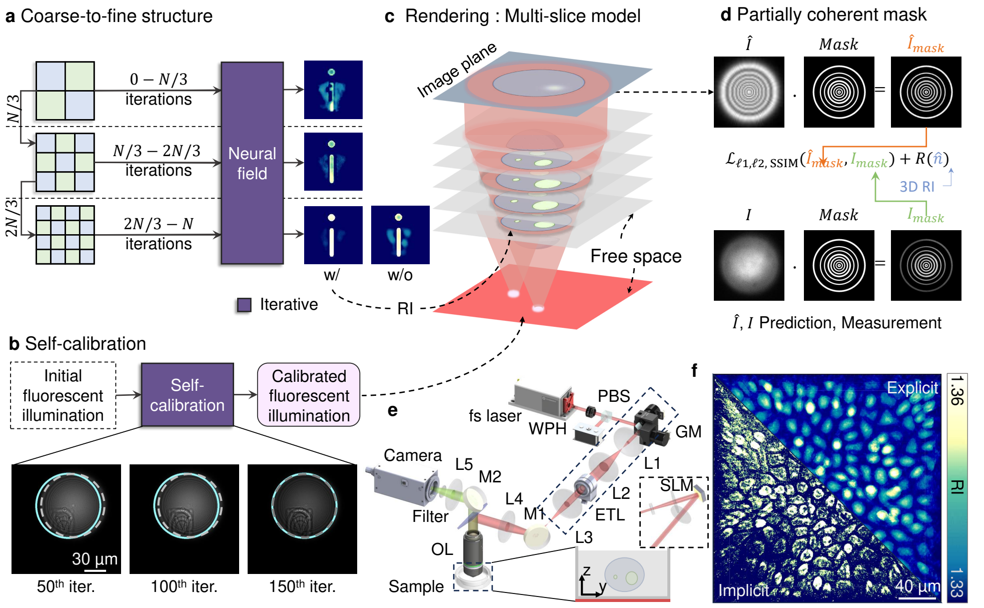

**Fig. 1 Overview of FDT using explicit neural fields. a-d** Reconstructing 3D RI from fluorescent images consists of four key components. **a**, The coarse-to-fine structure represents the unknown RI with explicit neural fields and resolve it through three stages with increasing resolution as the number of iterations increases to mitigate aliasing. **b**, Self-calibration adjusts the parameters to accurately model the fluorescent illumination for each measurement. These variables are then set as iterative parameters and optimized during the training process. **c**, The rendering equation is based on a differential multi-slice model, which takes the RI from **a** (shown as gray slices) and the fluorescent illumination sources from **b** (shown as two white spots on the bottom red slice) as inputs. The model calculates the light field as it is modulated by the heterogeneous RI on each slice using the Born approximate. Fresnel propagation is used to calculate light propagation between slices. **d**, A partially coherent light mask is applied to both the predicted and measured images to reduce the model mismatch. The masked images are used to calculate the loss function, incorporating $\ell_1$, $\ell_2$, Structural Similarity Index Measure (SSIM), and regularization terms. **e**, The optical diagram of FDT. A femtosecond (fs) laser is used for two-photon excitation. A 2-axis galvo mirror (GM) and the electrically tunable lens (ETL) on the conjugate back aperture enables random access to any 3D location. Excitation light is focused by an objective lens (OL) to focus on the sample, which consists of fluorophores on the bottom and label-free cells on top. Diffracted fluorescence is captured on a defocused plane by a camera. **f**, Comparison of the reconstructed 3D RI of living MDCK cells using FDT with (left) implicit neural fields representations and (right) explicit neural fields representations (ours), respectively. The explicit neural fields achieve significantly improved reconstruction quality.

the reconstructed RI to the ground-truth RI from $1.82 \times 10^{-6}$ to $1.57 \times 10^{-6}$ (Table S1).

Second, we develop a self-calibration method (Fig. 1b) that accurately calculates the illumination angle from each fluorescent illumination source, enabling highly precise forward rendering. The illumination angle is determined by the original position of the fluorescent source, the lateral resolution of the sampling grid, the distance between the source and the phase objects ("free space"), the number of $z$-planes and the distance between adjacent $z$-planes. These variables are set as iterative parameters and jointly optimized with the 3D RI. As the number of iterations increase, the position of illumination sources is fine tuned until the illuminated area (dash white circle) at the image plane in the predicted fluorescence image completely aligns with the illumination area (blue solid circle) from the ground-truth image (Fig. 1b). Compared to rendered images without self-calibration, the self-calibration improves the SSIM of the images from 0.9723 to 0.9933 and reduces the mean-square-error (MSE) of RI from $2.7980 \times 10^{-5}$ to $3.1984 \times 10^{-6}$ within 20 minutes (details in Methods 1, Table S1, and Fig. S1).

Third, both the estimated 3D RI from the first step and the fluorescent illumination angles from the second step are input to a differentiable multi-slice model (Fig. 1c) to generate the estimated forward fluorescence images. The multi-slice model treats the 3D RI of a bulky tissue as a stack of 2D slices that modulates the phase of input light from the slice below (the first slice modulates the light from fluorescent illumination) [12] (equations in Methods 1). The multi-slice model is integrated into the neural network framework so that the parameters of the forward rendering, the explicit neural fields, and the self-calibration are jointly optimized. By leveraging the flexible training framework, we implement batch training and dynamically adjust the learning process to enhance the generalization and training speed. To simultaneously solve the 3D RI and perform self-calibration, we assign different learning rates to the parameters involved in self-calibration and activate the self-calibration process during the second coarse-to-fine stage to prioritize RI reconstruction. This rendering step outputs estimated diffracted fluorescence images to be compared with the measured ones in the next step.

Fourth, to minimize the discrepancy between the incoherent/partially coherent fluorescence in the experimentally captured images and the coherent light used in the multi-slice model, we designed a partially coherent mask to filter both the predicted and measured image before calculating the loss (Fig. 1d). The partially coherent mask is an intensity mask generated by binarizing the coherent illumination at the image plane without phase objects. By computing the dot product of the partially coherent mask with the predicted image ($\hat{I}$) and the measured image ($I$), respectively, we generate the intensity-weighted predicted images ($\hat{I}_{mask}$) and the intensity-weighted measured images ($I_{mask}$). We then compute $\ell_1$, $\ell_2$, and SSIM losses between $\hat{I}_{mask}$ and $I_{mask}$ with a total variance (TV) regularizer to the predicted RI along all three dimensions (details in Supplementary 1.4). The partially coherent mask removes mismatching pixels from the loss calculation to ensure accurate reconstruction and quick convergence.

To compare our model to the state-of-the-art methods using implicit neural fields, we also constructed FDT with implicit neural representations following established methods [34, 37]. We first encode the coordinates into a high-frequency space, and then feed them into a 6-layer multi-layer perceptron (MLP) to generate the corresponding RI. We compared the reconstructed RI of MDCK cells from experimentally captured images using these two methods respectively (Fig. 1f), where FDT with the explicit model shows more accurate reconstruction compared to that reconstructed using the implicit model. More examples are in the next section. In summary, our model integrates the coarse-to-fine structure, self-calibration, differentiable multi-slice neural network model, and partially coherent mask to achieve high fidelity reconstruction of 3D RI from diffracted fluorescence images.

### Model validation with simulations and experiments using "UCDavis" patterns and microspheres of known RI

We first validate FDT by reconstructing the 3D RI of a simulated "UCDavis" pattern (Fig. 2a-e). The ground-truth RI model of the "UCDavis" pattern consists of 14 layers spaced 3 $\mu$m apart, sampled on a 512 × 512 grid with 0.33 $\mu$m pixel resolution. Each letter of "UCDavis" is centered in the odd-numbered layers, while even-numbered layers are homogeneous (Fig. 2e, top row). The RI is set to 1.38 for the letter and 1.33 for the background, approximating the RI of biological cells and culture media [46]. We set 400 illumination sources (a 20×20 array with 4.2 $\mu$m spacing) 60 $\mu m$ below the bottom layer of the "UCDavis" pattern and generated 400 ground-truth fluorescence images using the ground-truth RI under these illuminations (one represented image is shown in Fig. 2b). All images are captured on the same image plane, corresponding to the top layer of the "UCDavis" pattern. Our model is trained on the 400 ground-truth fluorescence images to reconstruct the 3D RI (Fig. 2a, e, bottom raw) and predict the corresponding fluorescence images (one represented predicted image is shown in Fig. 2c), following the procedure described in Fig. 1 and Section 1. The reconstructed RI (Fig. 2a) clearly resolves each letter with sharp edges and no crosstalk between adjacent $z$-layers. The predicted forward image (Fig. 2c; zoomed-in view on the bottom right) closely matches the ground-truth image (Fig. 2b; zoomed-in view on the top right), achieving a MSE of $2.0388 \times 10^{-6}$, a SSIM of 0.9994, and a PSNR of 56.9062, indicating a successful reconstruction. The reconstructed RI of each letter closely matches the ground-truth RI (Fig. 2e). Detailed quantitative evaluation is provided in the supplementary Table S1. The excellent $z$-sectioning ability of FDT is because it fills the "missing cone" in the optical transfer function (OTF) of a standard ODT (Fig. 2d) by using partially coherent/incoherent fluorescence illumination. The point-source nature also enables capturing diffracted light from multiple illumination angles in each image, whereas standard ODT captures diffraction from one illumination angle at a time. In summary, the result demonstrates that our model can successfully reconstruct the RI of overlapping phase objects along the $z$-axis with excellent optical sectioning.

To further validate FDT under realistic conditions, we next reconstruct the 3D RI of silica beads (RI = 1.46 at 635 nm) embedded in PDMS (RI = 1.41 at 635 nm) on a microscope slide pre-coated with a homogeneous fluorescence layer (central wavelength 635 nm), located 180 $\mu m$ beneath the beads. Using 419 experimentally captured fluorescence images, we reconstruct the 3D RI of a 5 $\mu m$ silica bead with both explicit and implicit neural field models and compared the results to simulations (Fig. 2f-h). Reconstructing silica beads using FDT is challenging due to self-focusing effects induced by the bead. Nonetheless, the results show explicit neural fields outperform implicit ones in both accuracy and speed. With experimentally captured images (SNR = 60, Fig. 2h, top left), the explicit neural fields yield homogeneous and aliasing-free reconstructions (Fig. 2f, 3rd column), while implicit neural fields show strong aliasing and missing features (Fig. 2f, 1st column). Minor elongation in the $z$ direction is attributed to the finite number of measurements and model mismatch. Using simulated

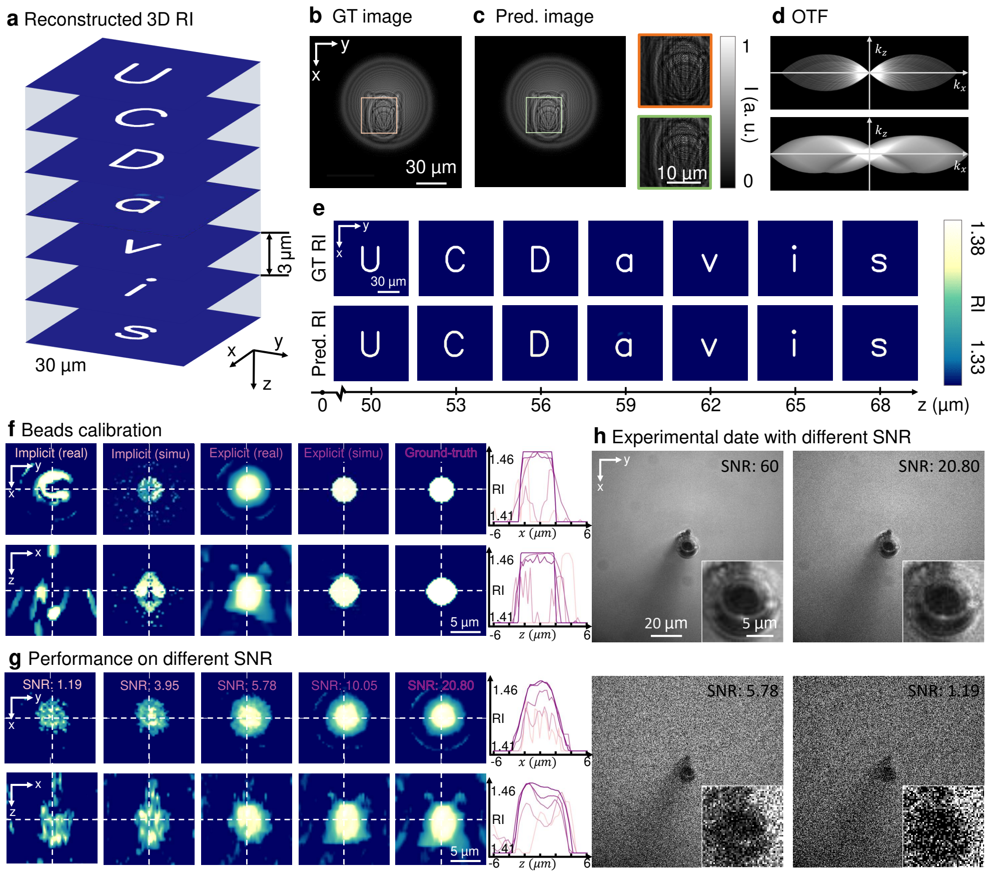

**Fig. 2 Reconstruction of the 3D RI of a simulated "UCDavis" pattern. a,** Reconstructed 3D RI distribution of the "UCDavis" pattern in 3D. **b,** An representative ground-truth (GT) image. **c,** Predicted fluorescence image together with zoomed-in views of the regions highlighted by the orange and green boxes. The MSE between the GT and predicted images and RI are $6.1125 \times 10^{-7}$ and $1.5730 \times 10^{-6}$, respectively. **d,** Compared to the optical transfer function (OTF) of conventional ODT (top), the OTF of FDT (bottom) solves the "missing cone" problem by extending coverage along the $k_z$-axis through point-source fluorescence illumination. **e,** Comparison of the ground-truth RI (top row) and the predicted RI (bottom row) on each $z$-plane, as indicated by the axis below. **f,** Calibration on silica beads using both simulated and experimental data. From left to right: implicit neural fields (experiment), implicit neural fields (simulation), explicit neural fields (experiment), explicit neural fields (simulation), ground truth (simulation). The dotted lines in the images mark the $x$- and $z$-directions for which the line profiles are plotted. **g,** Reconstruction performance at various signal-to-noise ratios (SNR), ranging from 1.19 down to 20.80. The dotted lines again indicate the $x$- and $z$-directions used to extract the line profiles. SNR = 5.78 is a boundary case: at or above this level, explicit neural fields preserve the bead shape accurately, whereas below it, reconstruction quality degrades significantly. **h,** Forward images of the same beads at different SNR levels. The top-left image (SNR=60) is used for the reconstruction in **f**, while the other three images are used for the reconstruction in **g**.

data, explicit neural fields reconstruct RI distributions nearly identical to ground truth (Fig. 2f, 4th vs. 5th column) from 600 diffracted fluorescence images, whereas implicit model shows aliasing and anisotropy (Fig. 2f, 2nd column). Line profiles across $x$ and $z$ (Fig. 2f, 6th column) confirm the explicit model reconstructs shaper edges and broader spatial frequency. Furthermore, reconstruction time is also reduced from 3 hours using the implicit model to 50 min using the explicit model (with 90% accuracy achieved in just 20 min). A quantitative comparison between the explicit model and the implicit model is provided in Table S2. In summary, these results quantitatively demonstrate that FDT with explicit neural fields enables accurate, efficient, and near isotropic 3D RI reconstruction, significantly improving performance over implicit neural fields.

To evaluate how the SNR of diffracted fluorescence images affects reconstruction quality, we test the robustness of FDT with explicit neural fields using experimental images across a range of noise levels (SNR 1.19-20, Fig. 2h), generated by adding Gaussian noise to a high SNR image (SNR = 60, Fig. 2h). The results show that FDT can accurately reconstruct the 3D RI when the image SNR is 5.78 or higher (Fig. 2g). In this regime, the bead's shape is well preserved and the RI is correctly reconstructed. Below this threshold, reconstruction quality degrades noticeably. Notably, even under severe noise, explicit neural fields outperform implicit ones with high SNR images (Fig. 2f, 1st column), providing higher overall fidelity. These results confirm that, despite relying on relatively dim fluorescence images compared to standard ODT using bright field transmission images, FDT with explicit neural fields can achieve accurate RI reconstruction, offering a unique advancement in integrating fluorescence imaging with quantitative phase reconstruction.

### Evaluation with experimental data of biological samples

In this section, we evaluate FDT by reconstructing the 3D RI of real biological samples, including 2D cultured living MDCK cells and 3D cultured bovine myotube. These label-free cells are cultured on a coverslip-bottom dish with fluorescent dye sprayed on the outside. Diffracted fluorescence images are captured at each of 441 excitation points arranged in a 21×21 grid ($8\mu m$ spacing) on a single imaging plane. The central wavelength of the fluorescence sources is 635 nm. Detailed information on sample preparation and the optical setup is provided in Section 1.3.

#### *3D RI reconstruction and analysis of living MDCK cells*

We first experimentally validate FDT using a thin layer of living MDCK cells (Fig. 3). The fluorescence sources are located 105 $\mu m$ below the MDCK cells (measured by self-calibration). All fluorescent images are taken on a single image plane slightly above the MDCK cells. A total of 390 fluorescence images are selected for reconstructing the 3D RI of the MDCK cells. The reconstructed RI (Fig. 3a) consists of 22 slices with a sampling grid of 1024 × 1024 pixels, forming a volume of $358.4 \times 358.4 \times 44\ \mu m^3$. The reconstructed RI ranges from 1.33 to 1.36, demonstrating FDT is capable of quantitatively reconstructing 3D RI with high sensitivity and over a large field-of-view. With the reconstructed RI, the predicted forward image (Fig 3b, zoomed-in view in Fig. 3c) successfully reconstruct the distinctive intracellular structures of the

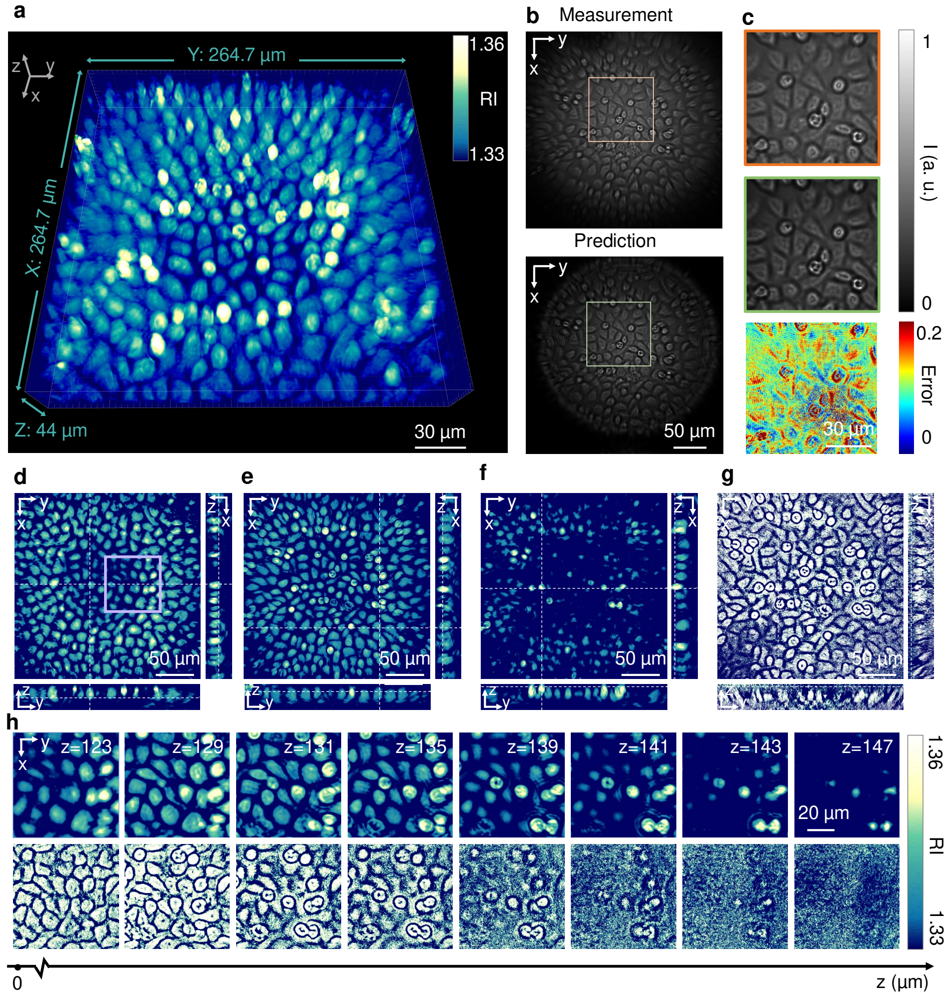

**Fig. 3 3D RI reconstruction of a thin layer of live MDCK Cells sample. a**, 3D visualization of the RI distribution of MDCK cells within a 358.4 × 358.4 × 44 $\mu m^3$ volume. The RI of the cells ranges between 1.33 to 1.36. **b**, Comparison of the measured (top) and predicted (bottom) images. **c**, Zoomed-in views of the highlighted region: the measured image (top), the predicted image (middle), and the error map between the measured and predicted images (bottom). **d-f**, Cross-sectional views of the RI distribution of MDCK cells on three representative planes that are 12.5 $\mu m$ apart, showing optical sectioning ability and high 3D resolution. **g**, Cross-sectional RI slices reconstructed using the implicit neural fields at the same location with **e** for direct comparison, which shows high-frequency artifacts and severe crosstalk from adjacent planes. **h**, Zoomed-in view of the image $z$-stack of cells in the boxed region in **d** reconstructed by the explicit model (top row) and the implicit model (bottom row). The $z$-position of each image is labeled on the $z$ axis below the images. The images again highlight the optical sectioning and high resolution of FDT using explicit neural fields. See the Supplementary Video for a better 3D visualization.

cells, as shown in the measured image. The pixel-wise error map (Fig. 3c, bottom) shows that the differences between the measured and predicted images are minimal. In the predicted image, regions where cells have detached from the substrate show relatively larger errors, while regions where cells remain adherent to the substrate are more accurate. These accurate forward images are fundamental to the successful RI reconstruction. The representative cross-sectional views of the RI in the $xy$, $xz$, and $yz$ planes (Fig. 3d-f) reveal individual cells clearly, highlighting FDT's ability to achieve high resolution in 3D and excellent $z$-sectioning ability. In the zoomed-in view of the image stack along the $z$ axis (Fig. 3h), cells can be observed appearing and disappearing. Most cells are alive and attached to the bottom of the dish (Fig. 3e-f), while in the top several layers (Fig. 3h, top row), dead cells shrink, disassemble their focal adhesions, and float above the substrate. The relatively higher RI of dead cells compared to live cells probably indicates changes in intracellular organelles during cell death, such as the condense of chromatin. Moreover, we compare FDT using explicit neural fields with FDT using implicit neural fields (Fig. 3g; Fig. 3h, bottom row). The implicit approach exhibits severe crosstalk from adjacent planes and high-frequency artifacts, consistent with the types of reconstruction artifacts observed in the silica beads experiment. These results experimentally and quantitatively demonstrate the strength of our method in achieving aliasing-free imaging with superior $z$-sectioning capability, high resolution, and high sensitivity.

During the reconstruction process, partially coherent masks are applied to mitigate the mismatch between experimental data and the simulation model. To evaluate their effectiveness, we quantitatively compare the experimentally measured images with the predicted images, both with and without the masks (Fig. S2, Table S3). Although both approaches enable the reconstruction of the 3D RI of MDCK cells, the reconstruction using partially coherent masks is more accurate, as indicated by the comparison between the predicted and measured images (Fig. S2). Quantitatively, without the masks, the mismatch in the fluorescence illumination field due to light coherence results in a relatively inaccurate reconstruction, with an MSE of $1.3453 \times 10^{-3}$. After applying the masks in the training process, the model mismatch is reduced, leading to a decrease in MSE to $6.2900 \times 10^{-4}$. Besides pixel-wise MSE, we also quantitatively compared other metrics, such as structural similarity (Table S3). The SSIM improves significantly after applying the mask, increasing from 0.8177 to 0.9160. In addition, despite the predicted image being synthesized under coherent illumination and the measured image under partially coherent illumination (Fig. 3b-c), the high accuracy of the RI reconstruction after applying the partially coherent masks makes these two images very similar. In conclusion, partially coherent masks provide an effective and efficient approach to handling partially coherent illumination in FDT.

#### *3D RI reconstruction and analysis of a bulky 3D cultured bovine myotube*

We next evaluate FDT by reconstructing the RI of a 3D cultured bovine myotube with complex structures (Fig. 4). Unlike the thin layer of cells discussed in the previous section, the bovine myotube is significantly thicker (about 300 $\mu m$, Fig. 4a) and consists of multiple layers of muscle cells. These muscle cells are differentiated from

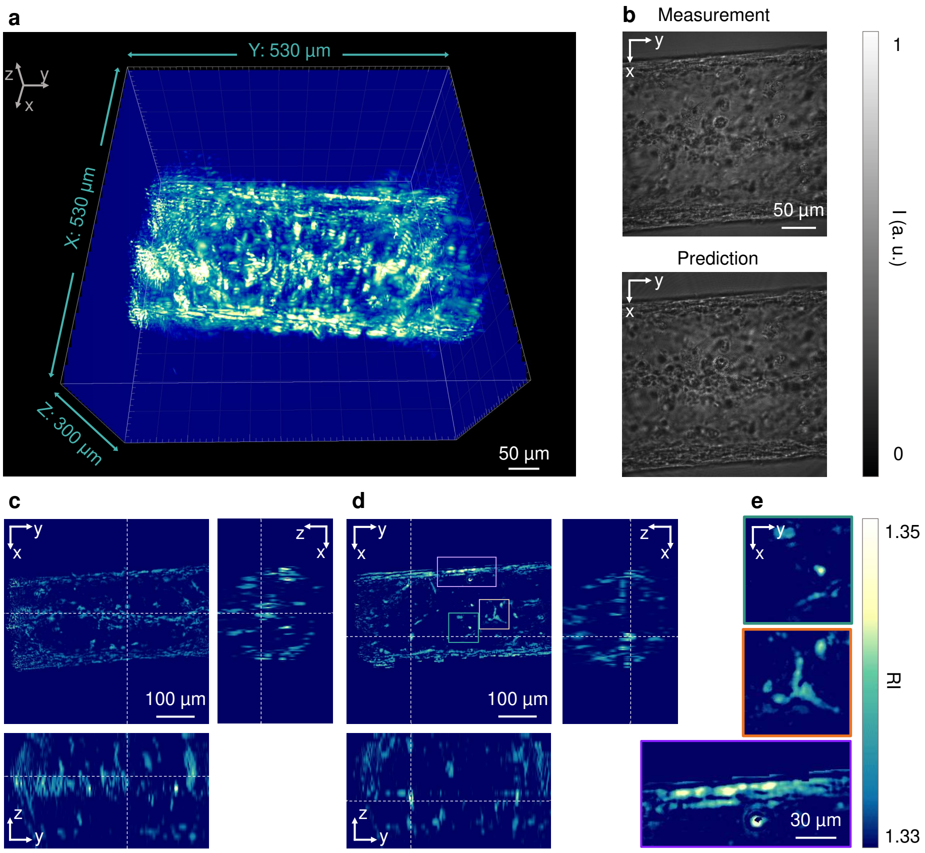

**Fig. 4 3D RI reconstruction of a 3D cultured bovine myotube.** **a**, 3D visualization of the RI of 3D cultured bovine myotube within a volume of $530 \times 530 \times 300$ $\mu m^3$. **b**, Comparison between the measured (top) and predicted (bottom) images of a representative region. **c-d**, Cross-sectional views of the reconstructed RI on two representative planes, showing high resolution and optical sectioning ability. **e**, Zoomed-in details of the highlighted regions in **d**, labeled by corresponding colors, showing different morphologies of stem cells in the 3D cultured bovine myotube during proliferation and differentiation. The results indicate that FDT can accurately reconstruct various structures across a wide range of spatial frequencies. See the Supplementary Video for a better 3D visualization.

label-free muscle stem cells cultured in 3D. Fluorescence sources are located 210 $\mu m$ below the myotube. Given that the bovine myotube is much thicker and more complex than the MDCK cells, the image plane is positioned 90 $\mu m$ below the top surface of the myotube to optimize the SNR of forward images rather than at the top of the sample. Correspondingly, the reconstruction process implements the back-propagation operation (Eq. 6) as well. After assessing the SNR of images, 348 fluorescence images are selected for 3D RI reconstruction in a $530 \times 530 \times 300$ $\mu m^3$ volume, consisting of 24 $z$-slices with a sampling grid of $1024 \times 1024$ pixels. Individual cells are not distinguishable in the scrambled forward images (Fig. 4b, top), but appear as bright and dark blurry shadows in the image depending on if they are above or below the image plane.

The predicted image (Fig. 4b, bottom) calculated with the reconstructed 3D RI closely matches the experimentally measured image, achieving a low MSE of $2.8607 \times 10^{-3}$. Cross-sectional views (Fig. 4c-d) show the reconstructed RI of the 3D cultured bovine myotube on two representative $z$-planes that are 50 $\mu m$ apart in the entire volume. These orthogonal views clearly reveal the tubular morphology of the sample. The circular cross-section in the $xz$ view confirms the integrity of the tube structure, while the clear delineation between the top and bottom of the tube highlights the effectiveness of our method's $z$-sectioning ability. The distinctive distribution of cells inside the tube on the two different $z$-planes also show the excellent $z$-sectioning ability of FDT. The reconstruction also achieves high 3D resolution. Individual cells are visible and distinguishable after reconstruction, whereas they are indistinguishable in the forward images. The reconstruction is sensitive to spatial RI variations of less than 1.5%, highlighting FDT's high sensitivity for quantitative phase imaging.

Our results reveal individual cells with diverse morphological structures and RI during the processes of differentiation and aggregation (Fig. 4e). This demonstrates that FDT not only achieves high resolution but also effectively reconstructs structures across both low and high spatial frequencies. The morphology of the muscle cells within the myotube can be categorized into three distinct differentiation stages: individual stem cells exhibiting membrane spreading (Fig. 4e, top; Fig. 4d, green box), interconnected cells forming pre-differentiation clusters within the matrix (Fig. 4e, middle; Fig. 4d, orange box), and elongated, stable muscle cells forming axial connections (Fig. 4e, bottom; Fig. 4d, purple box). The individual stem cells and the stable muscle cells exhibit a relatively higher RI compared to the interconnected cells, demonstrating RI as a potential biomarker to indicate the differentiation status of stem cells. Therefore, our method not only resolves cellular structures at high resolution but also quantitatively measures RI changes in cells at various metabolic states.

### Discussion

FDT is the first technique to enable 3D RI reconstruction of 300 $\mu m$-thick, label-free tissue using diffracted fluorescent images collected in reflection mode. FDT incorporates several key innovations in both hardware and software: (1) using partially coherent/incoherent fluorescent point-source as the illumination source is the key for optical sectioning; (2) defocused, diffracted fluorescence images allow 3D RI reconstruction from intensity-only images without a reference beam for interferometry; (3) explicit neural fields is a more interpretable model enabling faster and more accurate RI reconstruction, outperforming the state-of-the-art implicit neural fields; and (4) a combination of coarse-to-fine optimization, self-calibration, a differential multi-slice model, and partially coherent masks accelerates the reconstruction, mitigates model mismatching, and enables high resolution, high sensitivity, and aliasing-free reconstruction from realistic experimental images. We quantitatively validate FDT through simulations and experimental data of silica beads with known ground-truth RI, and then demonstrate its capability by reconstructing the 3D RI of living cells and bulky tissue volumes ($530 \times 530 \times 300$ $\mu m^3$) with subcellular resolution. FDT not only opens new opportunities for multimodal fluorescence-phase imaging, but also enables broad dissemination by leveraging standard two-photon microscopy hardware in a novel way, with a reconstruction algorithm that can be widely adopted for 3D rendering from multiple views.

Compared to our previous method, BRIEF [12], FDT enables faster and more accurate reconstruction of the RI in bulky, label-free tissues by incorporating explicit neural fields. Furthermore, by leveraging two-photon excitation, FDT overcomes the constrain of requiring sparsely distributed fluorophores, allowing broader, more realistic choices of fluorescence expression. We also benchmarked the performance of the explicit model against the implicit model used in the state-of-the-art IDT [37]. Our results demonstrate that the explicit model achieves substantially higher reconstruction fidelity, suppresses high-frequency aliasing artifacts, and reduces computational time. Finally, in contrast to conventional ODT systems that operate in transmission mode, FDT functions in reflection mode, which will facilitate future in vivo imaging applications.

FDT could be further optimized for broader applications. First, the current setup includes a spacer with a homogeneous RI (i.e., a coverslip) between the fluorescent sources and label-free biological samples. This spacer could probably be removed through further advancements in both the optical design and computational modeling. Second, the current implementation faces memory limitations, where the number of layers that can be processed is constrained by available GPU memory. This challenge may be addressed by developing memory-efficient algorithms to improve the scalability of the approach. Third, the current system relies on exogenous fluorescence as the illumination sources. While this is not a limitation for multimodal fluorescence-phase imaging, a fully label-free version of FDT could alternatively use intrinsic autofluorescence as the illumination source to accommodate applications where labeling is undesirable. Addressing these limitations would significantly enhance the practicality and applicability of FDT in a broad range of biological imaging scenarios.

### Conclusion

In summary, we present FDT as a new imaging modality capable of reconstructing the 3D RI of thick, label-free biological tissues using diffracted fluorescence images collected in reflection mode. FDT has the potential to enable in vivo multimodal fluorescence-phase imaging using standard two-photon microscopy hardware. This work lays the foundation for broadly accessible, high-resolution optical diffraction tomography in complex biological systems.

## Method

### Differential multi-slice model for 3D rendering

To improve computational efficiency and flexibility, we extend the conventional multi-slice model used in BRIEF [12] to the PyTorch framework. The differentiable multi-slice model with automated backpropagation mechanisms allows us to adjust the model's parameters arbitrarily. This flexibility enables fine-tuning of the RI resolution using the coarse-to-fine strategy and optimizing the self-calibration parameters for fluorescent illumination, as discussed earlier. In the multi-slice model, the 3D phase object is modeled as a stack of multiple $z$-layers, each with an unknown RI $\hat{n}_k(\mathbf{r})$, where $k = 1, 2, ..., N_z$. The 3D RI of a total of $N_z$ layers is:

$$\hat{n} \triangleq \{\hat{n}_k(\mathbf{r})\}_{k=1}^{N_z}, \quad \mathbf{r} = (x, y), \tag{1}$$

where $\mathbf{r} = (x, y)$ is the spatial coordinates. As light propagates through each layer, its phase is altered according to the transmission function $t_k(\mathbf{r})$ at $k^{th}$ layer:

$$t_k(\mathbf{r}) = \exp\left(\frac{j2\pi}{\lambda}\Delta z(\hat{n}_k(\mathbf{r}) - n_b)\right), \quad k = 1, 2, \ldots, N_z, \tag{2}$$

where $\lambda$ is the wavelength of the light, $\Delta z$ is the thickness of each layer, and $n_b$ is the RI of the background medium. We then use the operator $\mathcal{P}_{\Delta z}$ to represent the Fresnel propagation of the field over a distance $\Delta z$:

$$\mathcal{P}_{\Delta z}\{\cdot\} = \mathcal{F}^{-1}\left\{\exp\left(-j2\pi\Delta z\sqrt{\left(\frac{1}{\lambda}\right)^2 - ||\mathbf{k}||^2}\right) \cdot \mathcal{F}\{\cdot\}\right\}, \quad \mathbf{k} = (k_x, k_y), \tag{3}$$

where $\mathcal{F}\{\cdot\}$ and $\mathcal{F}^{-1}\{\cdot\}$ denote the Fourier transform and its inverse, respectively, and $\mathbf{k} = (k_x, k_y)$ is the spatial frequency coordinates. The electric field from the $i^{th}$ fluorescent source after propagating through the $k^{th}$ layer is described by:

$$\hat{E}_{k,i}(\mathbf{r}) = \mathcal{P}_{\Delta z}\left\{t_k(\mathbf{r}) \cdot \hat{E}_{k-1,i}(\mathbf{r})\right\}, \tag{4}$$

where $\hat{E}_{k-1,i}(\mathbf{r})$ is the electric field at the $(k-1)^{th}$ layer and $\hat{E}_{k,i}(\mathbf{r})$ is the electric field at the $k^{th}$ layer. If the image plane is at the top surface of the phase object (the final $z$-layer), the electric field at the camera under $i^{th}$ fluorescent illumination can be calculated by applying the pupil function $p(\mathbf{k})$ to the field at the final layer:

$$\hat{E}_i(\mathbf{r}) = \mathcal{F}^{-1}\left\{p(\mathbf{k}) \cdot \mathcal{F}\left\{\hat{E}_{N_z,i}(\mathbf{r})\right\}\right\}, \tag{5}$$

where $\hat{E}_{N_z,i}(\mathbf{r})$ is the electric field at the final layer. If the image plane is $\Delta Z_c$ below the final layer, as is the case when the phase object is thick, the field is first back-propagated over a distance $\Delta Z_c$ before passing through the pupil and arriving at the camera:

$$\hat{E}_i(\mathbf{r}) = \mathcal{F}^{-1}\left\{p(\mathbf{k}) \cdot \mathcal{F}\left\{\mathcal{P}_{-\Delta Z_c}\left\{\hat{E}_{N_z,i}(\mathbf{r})\right\}\right\}\right\}, \tag{6}$$

where $\mathcal{P}_{-\Delta Z_c}$ is the back-propagation operator. Since the camera can only detect the intensity of light field, the intensity image captured under $i^{th}$ fluorescent illumination is described as:

$$\hat{I}_i(\mathbf{r}) = |\hat{E}_i(\mathbf{r})|^2. \tag{7}$$

This mathematical model enables the simulation of the electric field's propagation through the layers of a sample within the PyTorch framework. However, the model assumes coherent light, while in experiments, two-photon excited fluorescence is partially coherent/incoherent light sources and spatially varying (depending on the local fluorescent label), leading to a mismatch between the model and actual experiments. We address this issue using a partially coherent mask.

### Details about the coarse-to-fine process

The coarse-to-fine process divides the training into three stages with a gradually increasing sampling grid in the $xy$ plane. In the simulation experiment of the "UCDavis" pattern, the total number of training iterations is 300. During the first 100 iterations, a coarse sampling grid of 128 $\times$ 128 pixels is used to model the unknown RI. For the subsequent 100-200 iterations, the sampling grid is increased to 256 $\times$ 256 pixels. Finally, during the 200-300 iterations, the highest sampling grid of 512 $\times$ 512 pixels is used. The number of $z$-layers remains at 14 throughout all three stage. At the transition points, bilinear interpolation is employed to upsample the RI to ensure smooth transitions between different sampling grids. Both experiments are trained using the Adam optimizer with a learning rate of $5 \times 10^{-3}$, and each training run takes approximately 20 minutes for 300 iterations.

### Details about the self-calibration of fluorescent illumination

In the simulated dataset of "UCDavis", the initial position $\hat{\boldsymbol{p}}_{\boldsymbol{i}} = (\hat{p}_{ix}, \hat{p}_{iy}, \hat{p}_{iz})$ is randomly draw from a Gaussian distribution $\mathcal{N}(0, \sigma^2)$ with uncertainty relative to the ground-truth position, defined as $\hat{\boldsymbol{p}}_{\boldsymbol{i}} = \boldsymbol{p}_{\boldsymbol{i}} + \mathcal{N}(0, \sigma^2)$. The ground-truth position $\boldsymbol{p}_{\boldsymbol{i}}$ is normalized to the range $[-0.5, 0.5]$, while the standard deviation $\sigma$ is set to be 0.1 to simulate the case where the initial estimation of the position is significantly different from the ground-truth. In the experimental data (Section 1), the initial position $\hat{\boldsymbol{p}}_{\boldsymbol{i}}$ is determined by fitting a 2D Gaussian function to the defocused fluorescence images. The mean of the 2D Gaussian represents the lateral position of the fluorescent source, while the standard deviation corresponds to the defocus distance in the $z$-axis. For one defocused fluorescence image $I_i$, the initial fluorophore position $\hat{\boldsymbol{p}}_{\boldsymbol{i}}$ is solved by:

$$\underset{\hat{p}_{ix}, \hat{p}_{iy}, \hat{p}_{iz}, A}{\operatorname{argmin}} \sum_{x=1}^{N} \sum_{y=1}^{N} \left\| I_i(x, y) - A \exp\left\{ -\frac{1}{2\sigma_z(\hat{p}_{iz})^2} \left[ (x - \hat{p}_{ix})^2 + (y - \hat{p}_{iy})^2 \right] \right\} \right\|_2^2 . \quad (8)$$

These positions are then set as iterative parameters that are updated continuously throughout the training process.

## Data availability

The data used for reproducing the results in the manuscript are available at FDT website

## Code availability

The code used for reproducing the results in the manuscript is available at FDT website

## Acknowledgement

Research reported in this publication was supported by the National Institute Of General Medical Sciences of the National Institutes of Health under Award Number R35GM155193. The content is solely the responsibility of the authors and does not necessarily represent the official views of the National Institutes of Health. This work was also supported by Dr. Yi Xue's startup funds from the Department of Biomedical Engineering at the University of California, Davis. We acknowledge Dr. Soichiro Yamada for providing the MDCK cells and biological support. We also acknowledge Dr. Jiandi Wan for providing the 3D cultured bovine myotube and mentoring Junjie Chen.

## Contributions

The project was conceived by Y.X., and R.H.. The code of the model was implemented by R.H.. The experiments were designed by R.H., Y.C., and Y.X.. The numerical results were collected by R.H.. The 3D cultured bovine myotube was cultured by J.C.. The data acquisition and preparation was conducted by Y.C. and R.H.. The manuscript was primarily drafted by R.H. and revised by Y.X., and reviewed by all authors.

## Competing interests

The authors declare no competing interests.

## Contents for Supplementary Material

**Supplementary Material** **22**

**1 Experimental setup** **22**
1.1 Optical setup used for imaging MDCK cells and 3D cultured bovine myotube . . . . . 22
1.2 Optical setup used for imaging silica beads . . . . . 23
1.3 Sample preparation. . . . . 23
1.4 Computational Setting . . . . . 24
1.4.1 Loss function. . . . . 24
1.4.2 Evaluation metrics. . . . . 25
1.4.3 Explicit representations used in FDT . . . . . 25

**2 Evaluation Metrics and Visual Analysis** **27**
Table S1: Quantitative results for self-calibration ablation study . . . . . 27
Table S2: Performance metrics for different reconstruction methods (polystyrene beads) . . . . . 27
Table S2: Performance metrics with/without partially coherent mask (MDCK cells) . . . . . 27
Fig. S1: RI reconstruction of the "UCDavis" pattern with and without self-calibration . . . . . 28
Fig. S2: Comparison of reconstructed results using FDT with and without partially coherent masks . . . . . 29

## 1 Experimental setup

### 1.1 Optical setup used for imaging MDCK cells and 3D cultured bovine myotube

The laser source for FDT is a femtosecond laser at 1035 nm wavelength and 1 MHz repetition rate (Monaco 1035-40-40 LX, Coherent NA Inc., U.S.). A polarizing beam splitter cube (PBS123, Thorlabs) and a half-wave-plate (WPHSM05-1310) mounted on a rotation mount (PRM05, Thorlabs) are used to manually adjust the input power to the following optics in the system. The laser beam is collimated and expanded by a 4-f system (L1, LC1120-C, $f_1$ = -100 mm, Thorlabs; L2, LA1256-B, $f_2$ = 300 mm, Thorlabs) before reaching a SLM (HSPDM1K-900-1200-PC8, Meadowlark Optics Inc., U.S.) placed on the Fourier plane. The SLM modulates the phase of the beam to selectively excite fluorophore at any given position in 3D. After the SLM, the laser beam is relayed by two 4-f systems (L3, LB-1889-C, $f_3$ =250 mm, Thorlabs; L4, AC508-250-C-ML, $f_4$ = 250 mm, Thorlabs; L5, AC508-200-C-ML, $f_5$ =200 mm, Thorlabs; L6, AC508-200-C-ML, $f_6$ = 200 mm, Thorlabs). Additionally, a black spot is painted on a mirror placed at the back focal plane of L3 to block the zero-order beam after the SLM. Next, the modulated laser beam is reflected by a dichroic mirror (FF880-SDI01-T3-35X52, Chroma, U.S.) to the back-aperture of the objective lens (XLUMPLFLN20XW, 20×, NA 1.0, Olympus Inc., Japan). Samples are placed on a manual 3-axis translation stage (MDT616, Thorlabs). In the emission path, a shortpass filter (ET750sp-2p8, CHROMA) is used to block the reflected excitation light. A bandpass filter (AT635/60m, CHROMA) is placed before the camera to pass through red fluorescence. The defocused fluorescence images are captured by a camera (Kinetix22, Teledyne Photometrics, U.S.) controlled by the Micro-Manager software. In addition, a one-photon widefield microscope is implemented, merged with the FDT setup, to locate the sample and find the focal plane before two-photon imaging. The one-photon system consists of an LED (M565L3, Thorlabs), an aspherical condenser lens (ACL25416U-A) to collimate the LED light, and a dichroic mirror (AT600dc, CHROMA) to combine the one-photon path to the two-photon path. The FDT system is controlled by a computer (OptiPlex 5000 Tower, Dell) using MATLAB (MathWorks, U.S.) and a data acquisition card (PCIe-6363, X series DAQ, National Instruments) for signal input/output. Customized MATLAB code is used to generate digital signals, and the NI-DAQ card outputs the signals to synchronously trigger the laser, the SLM, and the camera. For the MDCK cell experiment, the exposure time of each fluorescence image is 20 ms, and the total imaging time to collect 441 fluorescence images is 8.82 s. For the 3D myotube experiment, the exposure time of each fluorescence image is 100 ms, and the total imaging time to collect 441 fluorescence images is 44.1 s.

### 1.2 Optical setup used for imaging silica beads

The laser source for imaging silica beads is an optical parametric amplifier (Opera-F, Coherent NA Inc.) with a pumped laser at 1035 nm wavelength (Monaco 1035-40-40 LX, Coherent NA Inc., U.S.). The output laser beam wavelength is 880 nm. A polarizing beam splitter cube (PBS122, Thorlabs) and a half-wave-plate (WPHSM05-780) mounted on a rotation mount (PRM05, Thorlabs) are used to manually adjust the input power to the following optics in the system. A two-axis galvanometer system (GVS002, Thorlabs, Inc., USA) was integrated to implement a traditional point-scanning setup for actively modulating the excitation spot position within a single xy plane. Following the galvanometer, the laser beam is collimated and expanded by a 4-f system (L1, LA1401-B, $f_1$ = 60 mm, Thorlabs; L2, LA1979-B, $f_2$ = 200 mm, Thorlabs) before reaching an electrical tunable lens (ETL). The ETL (Optotune, EL-10-30-CI-NIR-LD-MV) is added at the Fourier domain to shift the focal point axially. The laser beam is relayed by two 4-f systems (L3, LA1979-B, $f_3$ =200 mm, Thorlabs; L4, LA1256-B, $f_4$ = 300 mm, Thorlabs; L5, AC508-200-C-ML, $f_5$ =200 mm, Thorlabs; L6, AC508-200-C-ML, $f_6$ = 200 mm, Thorlabs). Next, the modulated laser beam is reflected by a dichroic mirror (DMSP680B, Thorlabs, Inc.) to the back-aperture of the objective lens (XLUMPLFLN20XW, 20×, NA 1.0, Olympus Inc., Japan). The other parts of the setup, including the sample stage, detection path, and data acquisition parts, are the same as those in the optical setup used for imaging MDCK cells and 3D cultured bovine myotube.

### 1.3 Sample preparation.

**Silica microspheres in PDMS**. Silica beads (440345-100G, Sigma Aldrich Inc) with PDMS were prepared as a testing phantom. To prepare the polymer matrix,

PDMS base and curing agent were mixed at a weight ratio of 10:1. The glass beads, PDMS base, and curing agent were thoroughly combined and degassed for 30 minutes under vacuum to remove air bubbles. The resulting mixture was applied onto a fluorescence-coated glass slide. Fluorescence slides were prepared by uniformly spraying red fluorescent paint onto blank glass slides. A coverslip was gently placed on top of the PDMS mixture, and the entire assembly was cured in an oven at 70 °C for 2 hours.

**MDCK cells**. MDCK (Madin-Darby Canine Kidney) GII cells were cultured at 37°, 5% $CO_2$ in DMEM (Gibco) supplemented with 10% fetal bovine serum (FBS-RD Biosciences) and antibiotics. The cell line was checked for mycoplasma and routinely treated with mycoplasma removal agent (MRA) for preventive maintenance. The cells were plated onto a glass bottom dish (CellVis) pre-coated with rat tail collagen to promote cell adhesion to the substrate.

**3D cultured bovine myotube.** Bovine muscle stem cells were isolated from freshly slaughtered Angus cow semitendinosus muscle received from the UC Davis Meat Lab by adapting a previously reported protocol [47, 48]. Myotubes differentiated from the stem cells were fabricated employing an advanced Matrigel-agarose core-shell bioprinting technique. The filaments were subsequently cultured in Ham's F10 medium, enriched with 2% fetal bovine serum (FBS) and 1% penicillin-streptomycin (P/S). After 14 days, the well-differentiated bovine myotubes were carefully harvested from the filaments by dismantling the agarose shell.

### 1.4 Computational Setting

Our model is trained on an NVIDIA A6000 GPU with 48 GB of Memory. We use the Adam optimizer with an initial learning rate of $5 \times 10^{-3}$, a momentum decay rate of 0.9, and a decay rate of 0.99 for squared gradients. The batch size is set to 30.

#### 1.4.1 Loss function.

The loss function is designed to optimize multiple aspects of the model's performance by incorporating various components that address distinct error metrics and regularization terms. The formulation of the loss function is as follows:

$$\mathcal{L}(\hat{n}; I_1, I_2, \ldots, I_n) = \sum_{i=1}^{N_B} \mathcal{L}(\hat{n}; I_i), \tag{9}$$

where $\hat{n}$ is the predicted RI, $I_i$ represents the $i^{th}$ measurement of diffracted fluorescent image, and $N_B$ is batch size. The loss between each measurement and prediction is calculated as:

$$\mathcal{L}(\hat{n}; I_i) = \alpha\|\hat{I}_i - I_i\|_1 + \beta\|\hat{I}_i - I_i\|_2^2 + \gamma\ell_{\text{ssim}}(\hat{n}; I_i) + \tau_{xy}R_{xy}(\hat{n}) + \tau_z R_z(\hat{n}), \tag{10}$$

where $\hat{I}$ denotes the predicted images generated by our multi-slice model. Each term in the loss function is weighted by specific parameters ($\alpha$, $\beta$, $\gamma$, $\tau_{xy}$, and $\tau_z$) balance their contributions based on the model's objectives. These hyperparameters are carefully tuned to optimize performance for the specific application. The L1 loss, representing the mean absolute error, promotes sparsity in predictions, while the L2 loss, representing the mean squared error, helps minimize large deviations. Initially, $\alpha$ is set to 0 to focus on reducing large errors, and it is gradually increased during training to shift the emphasis towards sparsity and fine-tuning with $\beta$ decreases correspondingly, where $\alpha+\beta =$ constant. This dynamic adjustment allows the model to start by making broad corrections and then progressively refine its outputs, creating a robust framework for achieving high-quality predictions. Additionally, the SSIM loss is included to assess the similarity between predicted and ground-truth images, capturing perceptual differences and preserving image quality, as described below:

$$\ell_{\text{ssim}}(\hat{n}; I_i) = 1 - \text{SSIM}(\hat{I}_i, I_i), \quad \text{SSIM}(x,y) = \frac{(2\mu_x\mu_y + c_1)(2\sigma_{xy} + c_2)}{(\mu_x^2 + \mu_y^2 + c_1)(\sigma_x^2 + \sigma_y^2 + c_2)}. \tag{11}$$

Furthermore, to promote smoothness in the image along the $x$, $y$, and $z$ axes, we incorporate Total Variation (TV) regularization terms. Given the differing resolutions in the $xy$ and $z$ planes, we employ two separate weighting terms to control the contribution of these regularizers, which are defined as:

$$R_{xy}(\hat{n}) = \|\nabla_x \hat{n}\|_1 + \|\nabla_y \hat{n}\|_1, \quad R_z(\hat{n}) = \|\nabla_z \hat{n}\|_1. \tag{12}$$

#### 1.4.2 Evaluation metrics.

The evaluation metrics used in this study include MSE for measuring the average squared differences between predicted and actual values; SSIM for assessing perceptual similarity by considering luminance, contrast, and structure; Learned Perceptual Image Patch Similarity (LPIPS) for evaluating perceptual similarity based on deep features; and Peak Signal-to-Noise Ratio (PSNR) for indicating the peak error between images. These metrics collectively provide a comprehensive evaluation of the model's predictions in terms of both numerical accuracy and perceptual quality.

#### 1.4.3 Explicit representations used in FDT

State-of-the-art methods use implicit neural networks [37] to reconstruct the RI from intensity images, leveraging their ability to represent high-dimensional data. In our approach, the multi-slice model imposes a strong constraint on the RI. The multi-slice model enforces consistency across multiple 2D slices of the sample and ensures that the RI values are accurately aligned and correlated throughout the 3D volume. This inherent constraint reduces the complexity and ambiguity typically associated with high-dimensional data. Thus, instead of using implicit representations, we use explicit representations [33] to model the 3D RI (Fig. 1). Here, we provide more details about the framework (Fig. 5).

We first define a sparse voxel grid corresponding to the region of interest in the biological sample. Each voxel is then initialized as a network parameter using the Xavier method, representing the initial estimation of the unknown 3D RI. We then combine the explicit neural field with a coarse-to-fine structure, as described in Section 1, to adaptively upsample the grid as the number of iterations increases.

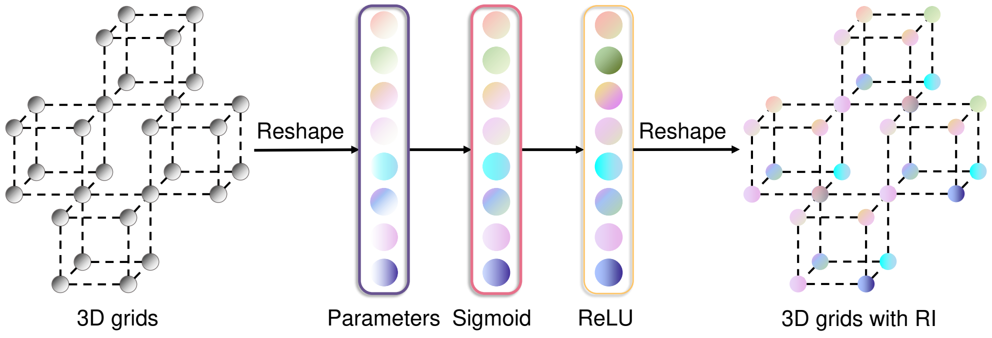

**Fig. 5 Structure of the explicit representation and neural field of FDT. a**, Initial 3D grids depicting interconnected nodes arranged in a spatial structure. **b**, Reshaping the 3D grids and setting them as the parameters of the neural field. **c**, Processing through parameter layers followed by sigmoid and ReLU functions. Mapping input values to a range between 0 and 1. **d**, Further reshaping the RI for the multi-slice model.

The explicit approach omits traditional neural network layers, such as linear or convolutional layers, which significantly accelerates the model's training speed. To prevent large or negative values, we apply a combination of sigmoid and ReLU activation functions to the parameters:

$$\text{Out} = \text{ReLU}(\text{Sigmoid}(x) - 0.5) \tag{13}$$

After this transformation, the parameters are reshaped back into grid format and then fed into the differential multi-slice model.

## 2 Evaluation Metrics and Visual Analysis

| Simulated data | | MSE↓ | SSIM↑ | LPIPS↓ | PSNR↑ |
|---|---|---|---|---|---|
| w/o coarse-to-fine | images | 3.2253e-06 | 0.9994 | 0.0004 | 54.9143 |
| | RI | 1.8282e-06 | 1.000 | 0.0010 | 57.3798 |
| w/ coarse-to-fine | images | 6.1125e-07 | 0.9999 | 0.0001 | 62.1378 |
| | RI | 1.5730e-06 | 1.000 | 0.0007 | 58.0327 |
| w/o self-calibration on parameters | images | 1.0876e-03 | 0.8317 | 0.0720 | 29.6355 |
| | RI | 7.7316e-05 | 0.9977 | 0.0063 | 41.1173 |
| w/ self-calibration on parameters | images | 5.6229e-06 | 0.9990 | 0.0006 | 52.5004 |
| | RI | 5.2851e-06 | 1.0000 | 0.0019 | 52.7695 |
| w/o self-calibration on positions | images | 1.1377e-04 | 0.9723 | 0.0065 | 39.4398 |
| | RI | 2.7980e-05 | 0.9994 | 0.0027 | 45.5315 |
| w/ self-calibration on positions | images | 3.1797e-05 | 0.9933 | 0.0052 | 44.9761 |
| | RI | 3.1984e-06 | 0.9999 | 0.0007 | 54.9507 |

**Table S1** Quantitative results from the ablation study on the coarse-to-fine structure and self-calibration using the simulated data "UCDavis". The "w/" and "w/o" coarese-to-fine conditions are evaluated without applying self calibration (i.e., using perfect illumination source positions). For the self-calibration study, random Gaussian noise is added to parameters related to the optical system and illumination positions.

**Table S2** Performance Metrics for Different Reconstruction Methods for simulated silica beads.

| Method | MSE | SSIM | LPIPS | PSNR |
|---|---|---|---|---|
| Implicit | $6.1572\ \times 10^{-3}$ | 0.8724 | 0.2363 | 32.0476 |
| Explicit | $5.6387\ \times 10^{-4}$ | 0.9834 | 0.0463 | 47.5725 |

**Table S3** Performance metrics with and without the partially coherent mask for MDCK cell reconstruction.

| Method | MSE | SSIM | LPIPS | PSNR |
|---|---|---|---|---|
| w/o mask | $1.3453\ \times 10^{-3}$ | 0.8177 | 0.1776 | 28.7119 |
| w/ mask | $6.2900\ \times 10^{-4}$ | 0.9160 | 0.1417 | 32.0135 |

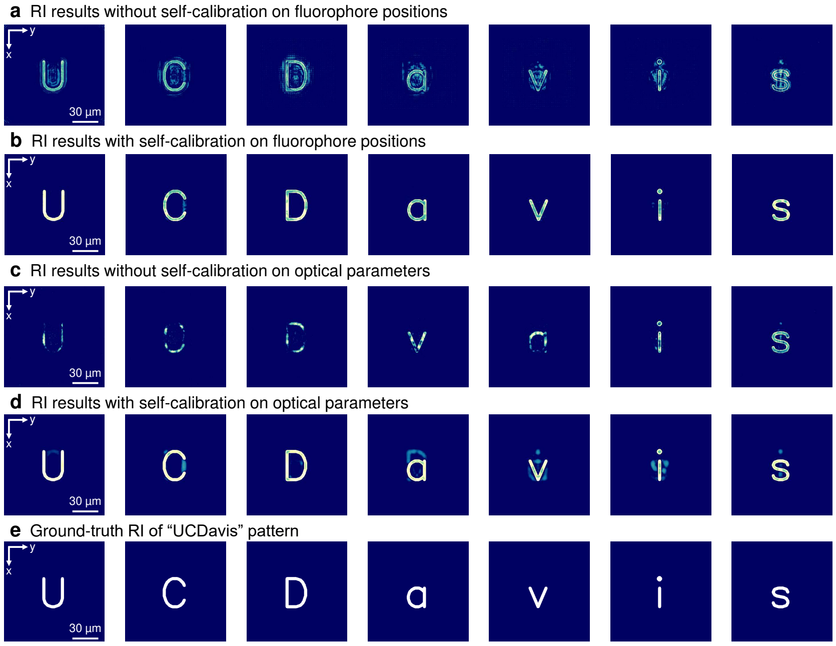

**Fig. S1 RI reconstruction of the "UCDavis" pattern with and without self-calibration.** **a**. Reconstructed RI without self-calibration on fluorophore positions shows significant distortion and noise. **b**. Reconstructed RI with self-calibration on fluorophore positions displays improved clarity and accuracy of the pattern. **c**. Reconstructed RI without self-calibration on optical parameters (e.g., the resolution of sampling grid) suffers from similar distortions seen in **a**. **d**. Reconstructed RI with self-calibration on optical parameters demonstrates further enhancement, closely matching the expected output. **e**. Ground-truth RI of the "UCDavis" pattern, showing the ideal pattern for comparison.

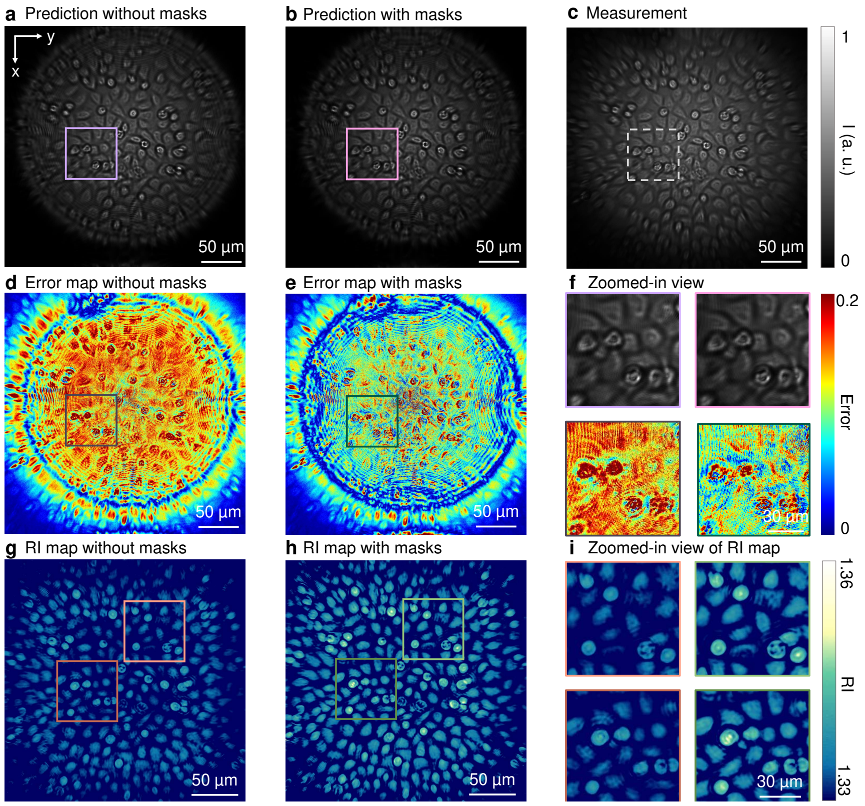

**Fig. S2 Comparison of reconstructed results using FDT trained with and without partially coherent masks. a-b**. Predicted image generated using the 3D RI calculated with FDT, trained **a** without and **b** with partially coherent masks. **c**. Experimentally measured image under the same illumination as in **a-b**. **d-e**. Error map between predicted image and measured images: **d** without and **e** with partially coherent masks. The partially coherent masks significantly reduce errors and improve accuracy. **f**. Zoomed-in view of representative regions in the images and error maps, confirming that the partially coherent masks improve the accuracy for both the bottom and top layer cells. **g-h**. A representative $z$-plane of the reconstructed RI: **g** without and **h** with the partially coherent masks, with 70 pixels cropped from each edge for better visualization. **i**. Zoomed-in view of representative regions in the RI maps.

## Supplementary Files

This is a list of supplementary files associated with this preprint. Click to download.

- mdckzsection.mp4
- mdckrotate.mp4
- 3dtuberotate.mp4
- 3dtubezsection.mp4
- mdckimplicitzsection1.mp4
- mdckimplicitrotate.mp4
